# Dysfunctional MDR-1 disrupts mitochondrial homeostasis in the oocyte and ovary

**DOI:** 10.1038/s41598-019-46025-x

**Published:** 2019-07-03

**Authors:** Haley Clark, Laura O. Knapik, Zijing Zhang, Xiaotian Wu, Mandar T. Naik, Nathalie Oulhen, Gary M. Wessel, Lynae M. Brayboy

**Affiliations:** 1 Department of Obstetrics and Gynecology, Division of Reproductive Endocrinology and Infertility, Women & Infants Hospital of Rhode Island, Alpert Medical School of Brown University, 101 Dudley Street, Providence, RI 02905 USA; 2 Alpert Medical School of Brown University, 222 Richmond Street, Providence, RI 02903 USA; 3 Department of Molecular Biology, Cell Biology and Biochemistry, Brown University, 185 Meeting Street, Providence, RI 02912 USA; 4 School of Public Health Brown University, 121 South Main Street, Providence, RI 02903 USA; 5 Brown University Structural Biology Core, 70 Ship Street, Providence, RI 02903 USA

**Keywords:** Energy metabolism, Oogenesis

## Abstract

Multidrug resistance transporters (MDRs) are best known for their pathological role in neoplastic evasion of chemotherapeutics and antibiotics. Here we show that MDR-1 is present in the oocyte mitochondrial membrane, and it protects the female gamete from oxidative stress. Female *mdr1a* mutant mice have no significant difference in ovarian follicular counts and stages, nor in reproductively functioning hormone levels, yet these mice are significantly more vulnerable to gonadotoxic chemotherapy, have chronically elevated reactive oxygen species in immature germinal vesicle oocytes, exhibit a significant over-accumulation of metabolites involved in the tricarboxylic acid cycle (TCA), and have abnormal mitochondrial membrane potential. The *mdr1a* mutant ovaries have a dramatically different transcriptomic profile with upregulation of genes involved in metabolism. Our findings indicate that functionality of MDR-1 reveals a critical intersection of metabolite regulation, oxidative stress, and mitochondrial dysfunction that has direct implications for human infertility, premature reproductive aging due to oxidative stress, and gonadoprotection.

## Introduction

Multidrug resistance transporters (MDRs) belong to a family of ATP Binding Cassette (ABC) transporters with 49 genes in the human^1^. These transporters are embedded in phospholipid bilayers and use ATP hydrolysis to efflux substrates and cytotoxic substances^2^. The most well studied of all ABC transporters is Multidrug Resistance Transporter 1 (MDR-1), also known as *ABCB1* or P-glycoprotein (P-gp). The gene is located on chromosome 7q21.12. It is a highly polymorphic gene with more than fifty single nucleotide polymorphisms^3^. In normal physiology, MDR-1 maintains epithelial barrier function in tissues such as the intestines, kidney, liver, and testis^4^. It has also been reported that the *ABCB1* (human MDR-1 gene) polymorphism *C3435T*, with allele frequency of 24.2% in the US population and 69.3% in the Ashkenazi Jewish population^5^ increases risk of many chronic diseases associated with oxidative stress such as Alzheimer’s Disease^6,7^, Inflammatory Bowel Disease^8^ and breast cancer^9,10^. Recent work has begun to reveal a link between abnormal MDR-1 expression and decreased mitochondrial functionality^11^. This link has been further fortified by the localization of MDR-1 to the mitochondrion^12^ and its subsequent upregulation mitigating oxidative stress in retinal cells^13^.

Recent work has highlighted the importance of MDRs in normal reproductive physiology^14^. However, a mechanistic understanding of MDR functionality, especially in the ovary, is still lacking. The expression of at least 30 MDR proteins is detectable in human and mouse ovaries, among which MDR-1 is particularly abundant. However, there is a dearth of information regarding the function of MDR-1 in the ovary and oocyte. Our group was the first to describe the expression and functionality of MDR-1 in murine and human oocytes^15^. Our subsequent research in *mdr1a/mdr1b/bcrp*−/− knockout animals showed that loss of these transporters caused metabolite accumulations potentially signaling increased oxidative stress^16^. This finding increased our focus on the interaction between MDR-1 and the mitochondrion in the oocyte.

There is a growing body of evidence indicating that MDR-1 localizes to the mitochondria in somatic cells. MDR-1 efflux may protect mtDNA from mutagens and mitotoxic substances^17,18^. The mitochondria are abundantly present and are thought to be particularly essential for the developmental potential of the oocyte^19^. The blastocyst is completely dependent on maternal inheritance of mitochondria from the oocyte^19^. There are data that demonstrate oocyte mitochondria generate low levels of ATP via mitochondrial aerobic respiration^20,21^. Oxidative phosphorylation leads to the generation of reactive oxygen species (ROS) and potentially ovotoxic byproducts. The presence of MDR-1 may facilitate the exportation of these byproducts and may be crucial for normal mitochondrial function in the oocyte. Here we investigated the role of MDR-1 in the maintenance of ovarian and oocyte metabolic homeostasis. We utilized a *mdr1a* mutant mouse strain (*mdr1a* mutant) that has been reported to express non-functional MDR-1, due to insertion of murine leukemia virus long terminal repeat (muLV-LTR) sequence in the *mdr1a* locus^21,22^. We chose the mutant with an inactivating mutation as a model, because it may more closely resemble human disease compared to a complete knockout of *mdr1a*.

In this paper, our primary goal was to understand how loss of functional MDR-1 affects the mitochondria of the oocyte and ovary. We have observed that the oocyte mitochondrion does express MDR-1, and that the ovary has markedly distinct functional features including increased oxidative stress, susceptibility to chemotherapeutics, and distinct gene expression and metabolic profiles.

## Results

### *mdr1a* mutant mice express aberrant MDR-1 protein

**Figure 1:**
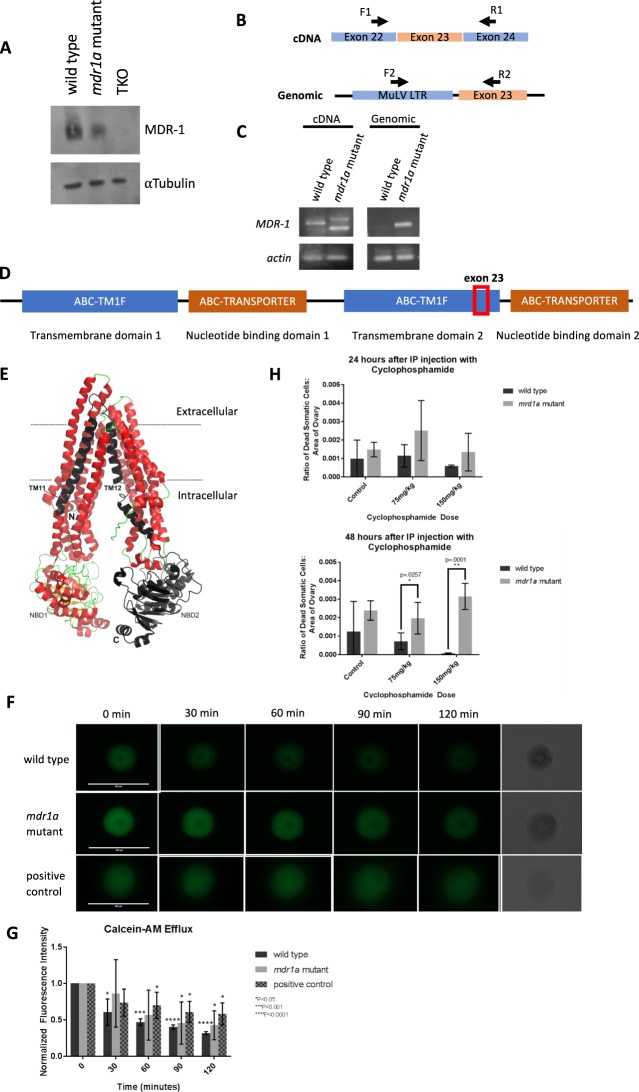
Characterization of *mdr1a* mutant. (**A**) Expression levels of MDR-1 in ovary lysates of wild type, *mdr1a* mutant, and *mdr1a/mdr1b/bcrp*−/− triple knockout (TKO) animals. Blots were cropped for display. Full-length blots are presented in (Fig. S6A). (**B**) Schematic of primers for the validation of the perturbation of *mdr1a* exon 23 in *mdr1a* mutants. Primers F1 and R1 were used to validate the perturbation of *mdr1a* exon 23 in the transcript (cDNA). Primers F2 and R2 were used to validate the insertion of MuLV LTR sequence in *mdr1a* locus (Genomic) in the genome of *mdr1a* mutant mice. (**C**) PCR amplification. With wild type ovarian cDNA template, F1/R1 amplified a 471 bp product (no perturbation). With *mdr1a* mutant cDNA template, F1/R1 amplified a larger product of ~500 bp (replacement of exon23 with a fragment of MuLV LTR sequence) and a smaller product of ~400 bp (deletion of exon 23). With wild type genomic DNA template, F2/R2 gave no amplification (absence of insertion). With *mdr1a* mutant genomic DNA template, F2/R2 amplified a ~550 bp product (MuLV LTR insertion). Actin was amplified as a positive control. Gels were cropped for display. Full-length gels are presented in (Fig. S6C,D). (**D**) Illustration of functional domains on MDR-1 protein. Blue represents transporter integral membrane type-1 fused domains. Orange represents ATP-binding cassette, ABC transporter-type domains. (**E**) Structure of mouse P-glycoprotein (PDB accession code: 3G5U). The region deleted by skipping of exon 23 is shown in black, including the second nucleotide binding domain (NBD2) and transmembrane helices TM11 and TM12. The two nucleotide binding domains, and N and C-terminals are labeled. The lipid bilayer is indicated by dotted lines. (**F**) Wild type and *mdr1a* mutant oocytes exhibit calcein-AM fluorescence from timepoint 0 to 4 (every 30 min), with positive control wild type oocytes incubated with 25 mM PSC 833. Scale bar: 200 µm. (**G**) Normalized fluorescence intensity (∆F/F) of the change in individual oocyte fluorescence intensity over time (n = 4). Mutant oocytes exhibit a slower decrease in fluorescent intensity compared to wild type oocytes. (**H**) Ovaries from animals treated with 75 mg/kg and 150 mg/kg cyclophosphamide show increases in cell death with LIVE/DEAD Cell Viability Assay at 24 hours. Cell death is most apparent in cells that morphologically appear to be somatic and not oocytes. *mdr1a* mutants display more cell death at 48 hours when exposed to cyclophosphamide compared to wild type controls.

In order to utilize the *mdr1a* mutant mice in our functional study of the MDR-1 protein, we first validated the presence of the *mdr1a* mutation in the *mdr1a* mutant mouse strain. We examined the *mdr1a* transcript and genomic sequence using primers surrounding exon 23 and the reported murine leukemia virus insertion (muLV-LTR insertion) (Fig. 1B). Consistent with the previous reports, *mdr1a* mutants express *mdr1a* transcripts with exon 23 either completely absent (Fig. 1C, cDNA: *mdr1a* mutant smaller product) or replaced by a fragment of muLV-LTR sequence (Fig. 1C, cDNA: *mdr1a* mutant larger product). The insertion of muLV-LTR is present in the *mdr1a* genomic locus of the mutant, but not that of the wild type (Fig. 1C, genomic).

Exon 23 is located close to the 5′ end of the transcript and encodes a part of the transmembrane domain in the MDR-1 protein (Fig. 1D)^23,24^. In order to understand the effect of exon 23 deletion or insertion of a viral peptide at that location, we analyzed previously solved crystal structure of mouse P-glycoprotein (Fig. 1E)^25^. It can be seen that the deletion of exon 23 will abolish the region shown in black, which not only includes the second nucleotide binding domain (NBD2), but also two transmembrane helices, TM11 and TM12. Thus, this truncated protein isoform will have reduced activity. Similarly, insertion of the viral sequence will disrupt folding and membrane topology of the protein that will drastically affect its function. As expected, the loss of exon 23 does not seem to disrupt the general expression of MDR-1 protein. We detected MDR-1 protein in the ovarian lysate of both the wild type and the *mdr1a* mutant, but not the *mdr1a/mdr1b/bcrp*−/− triple knockout mouse (Fig. 1A).

We tested MDR-1 functionality in the *mdr1a* mutant by comparing efflux capability in germinal vesicle oocytes from both wild type and mutants using an MDR-1 substrate called Calcein-AM (Fig. 1F). Normal cells efflux the non-fluorescent compound before endogenous esterase activity can cleave the acetomethoxy (AM) group causing calcein to fluoresce. Germinal vesicle oocytes were chosen given they have the most efficient efflux of all maturational stages as we have previously reported^15^. Here, the wild type oocytes exhibited a significant decrease in normalized fluorescent intensity after just 30 minutes and continue to decrease over time (Fig. 1G). The mutant oocytes, however, only exhibit a significant decrease in fluorescence at 90 and 120 minutes, and this decrease is not as great as exhibited in wild type oocytes. The mutant oocytes also displayed a wide range of fluorescence values. These findings indicate that MDR-1 in the *mdr1a* mutant animals does not efflux as wild type oocytes do.

### *mdr1a* mutants have increased sensitivity to cyclophosphamide

We then probed whether dysfunctional MDR-1 causes increased susceptibility of the ovary to toxicants. Since its discovery, MDR-1 has been notoriously characterized to eliminate toxic drugs and protect cancer cells from chemotherapy by effluxing cytotoxic drugs out of neoplastic cells. Cyclophosphamide is an alkylating chemotherapeutic agent and is known to be gonadotoxic^15,16,26,27^. We find significantly increased cell death *in vivo* of ovarian cells in the *mdr1a* mutant ovaries compared to wild type ovaries within only 48 hours of exposure (Fig. 1H). The somatic cells most susceptible to the toxicant were consistent with granulosa cell morphology and location, and long-term low-level exposures are hypothesized to result in compromised oocytes. These findings suggest that as observed in cancer cells, MDR-1 protein may play an important role in the removal of environmental toxicants from ovarian cells, and thus it may play an integral role in its detoxification.

### *mdr1a* mutant ovaries have an abnormal metabolic profile

**Figure 2:**
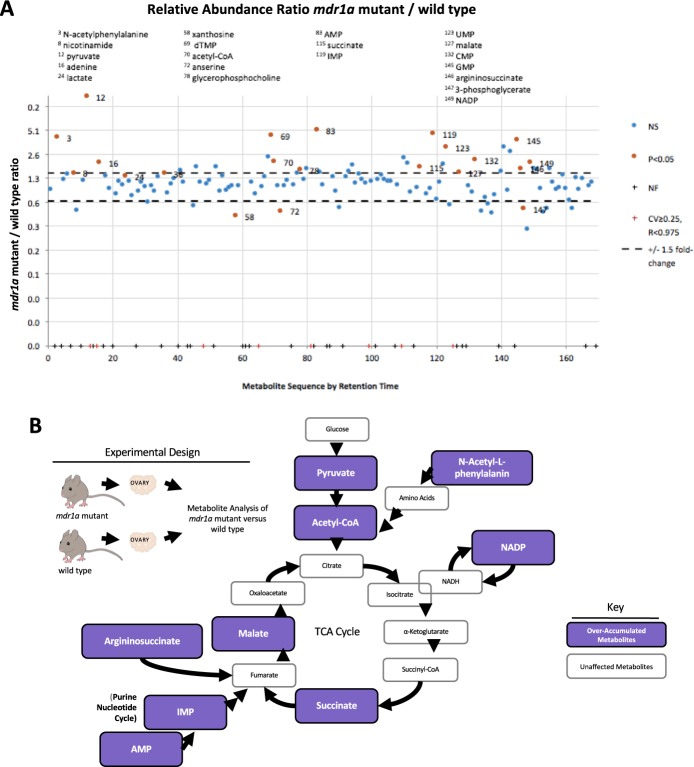
TCA metabolite over-accumulation indicates mitochondrial dysfunction in mdr1a mutant ovaries. (**A**) *mdr1a* mutant ovaries have over accumulation of metabolites in the Krebs Cycle. (**B**) Ovaries from *mdr1a* mutant and wild type mice were analyzed for polar and non-polar metabolites. Thirteen metabolites showed significant over-accumulation in *mdr1a* mutant mouse versus wild type, which are indicated in purple above. Nine of the thirteen were associated with the TCA cycle. The other metabolites were adenine, GMP, dTMP and CMP.

Analysis of 169 polar metabolites was performed on both wild type and *mdr1a* mutant ovaries (three replicates from different individual animals for each genotype). From this widely dispersed candidate pool, 13 metabolites showed significant overabundance in the mutant ovaries when compared to the wild type controls (Fig. 2A). The following metabolites that were found to be significantly different between wild type and *mdr1a* mutant ovaries include (with p-values) pyruvate (p = 0.032), acetyl-CoA (p = 0.048), nicontinamide adenine dinucleotide phosphate (NADP^+^ the reduced form in NADPH) (p = 0.006) N-N-acetylphenylalanine (p = 0.019), adenine (p = 0.016), succinate (p = 0.019), arginosuccinate (p = 0.019), malate (p = 0.038), dTMP (p = 0.048), AMP (p = 0.016), IMP (0.024), CMP (p = 0.025), and GMP (p = 0.019). Remarkably, 9 of the 13 metabolites are products of the TCA cycle and suggest that *mdr1a* may be an upstream regulator in this critical pathway essential to oxidative phosphorylation.

Perhaps more surprising in these analyses were three metabolites that showed lower accumulation in mutant ovaries compared to wild type (p < 0.05). The metabolites with decreased levels relative to the wild type included anserine (p = 0.045), xanthosine (p = 0.015), and 3-phosphoglycerate (p = 0.037) (Fig. 2A).

### *mdr1a* mutant ovaries show distinct transcriptomic profiles with an upregulation of genes involved in metabolism and in the mitochondrion

**Figure 3:**
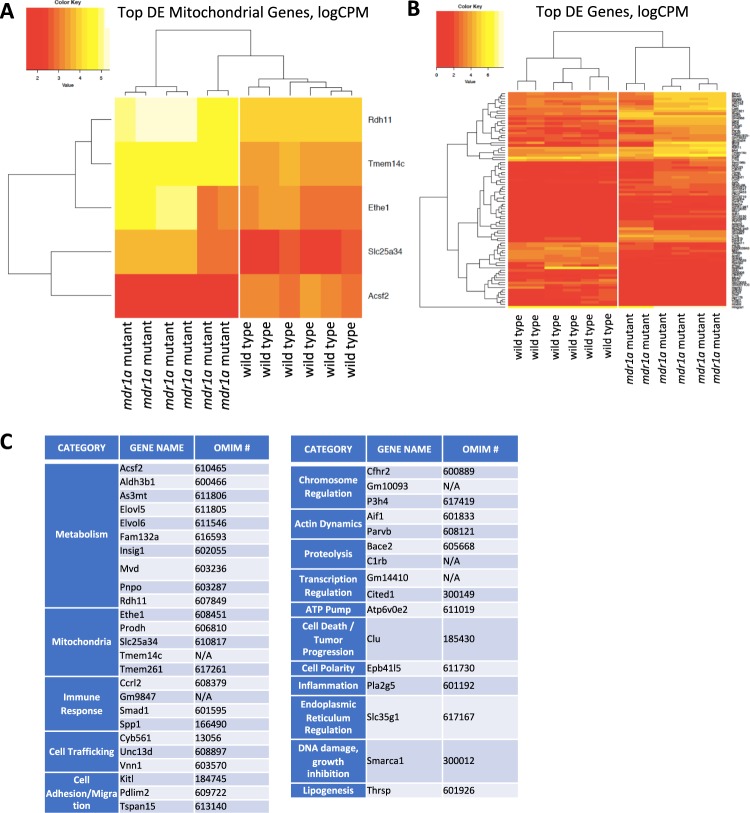
Ovarian transcriptome of *mdr1a* mutants reveals upregulation of genes involved in metabolism and mitochondria. Many of the genes that are differentially expressed between the wild type and the mutant are those that code for genes involved in metabolism as well as mitochondrial genes. (**A**) Heat map of mitochondrial genes (**B**) Heat map of the top 50 differentially expressed genes. Yellow, high value; red, low value; DE, differential expression; CPM, counts per million. (**C**) This table represents the top categories of genes that were differentially expressed between *mdr1a* mutant and wild type ovaries. Note the largest categories are metabolism and mitochondria.

In order to further dissect the impact of MDR-1 mutation in the ovary, we analyzed and compared the transcriptomic profile between the wild type and *mdr1a* mutant ovaries. Six ovaries from age-matched individual animals of *mdr1a* mutant and wild-type genotypes were processed for differential gene expression (differential RNA-seq). Remarkably, 798 mRNAs showed statistically significant differential steady state levels out of the approximately 16,000 genes expressed in a mouse ovary. The top 50 differentially expressed known genes between wild type and *mdr1a* mutant mice are highlighted (Fig. 3B). Of those with known function, the majority of the genes are involved in metabolism and mitochondrial homeostasis (Fig. 3C**)**. Thus, as for the metabolites, the MDR-1 mutation does not wholesale increase/decrease mRNA accumulations, and instead, a diverse set of gene products involved chiefly in metabolism appears to be differentially regulated with and without a functional MDR-1. We further note that the consistency of the vast majority of the mRNAs within a cohort is strong. We further validated our RNA Seq data by performing qPCR with the top differentially expressed genes: *acsf2*, *c1rb*, *mid1*, *zbtb8b*, *gulo*, *tspan11*. *c1rb* showed a 2-fold increase in expression in the mutant, while the remaining genes showed a drastic decrease in expression in the mutant, compared to wild type controls (Fig. S1).

### *mdr1a* mutant oocytes have increased oxidative stress

**Figure 4:**
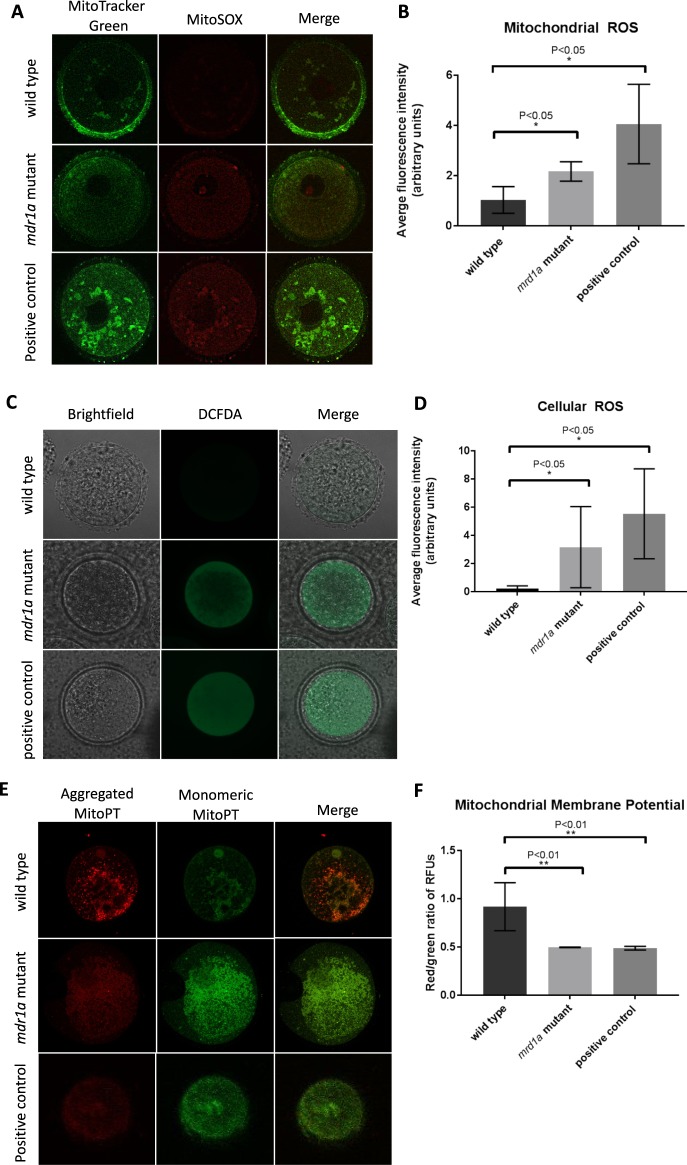
*mdr1a* mutant oocytes have increased ROS and perturbed mitochondrial membrane potential. (**A**) Images of oocytes from *mdr1a* mutant and wild type mice stained with MitoSOX Red Mitochondrial Superoxide Indicator imaged imaged on a confocal microscope (60x oil magnification) to show MitoTracker Green, MitoSOX, and merge. (**B**) Quantification of average fluorescence intensity (wild type n = 4, *mdr1a* mutant n = 8, positive control n = 4). Significantly more (p < 0.05) mitochondrial superoxide signaling was detected in the *mdr1a* mutant oocytes compared to wild type oocytes at baseline without a toxic challenge. (**C**) Images of wild type, *mdr1a* mutant, and wild type H_2_O_2_ treated oocytes as a positive control stained in CM-H_2_DCFDA imaged on a confocal microscope (40x oil magnification) in brightfield, GFP, and merge. (**D**) Quantification of average fluorescence intensity (wild type n = 5, *mdr1a* mutant n = 9, positive control n = 5). Significantly more (p < 0.05) cellular superoxide signaling was detected in the *mdr1a* mutant oocytes compared to wild type oocytes. (**E**) Images of wild type, *mdr1a* mutant (60x oil magnification), and wild type CCCP treated oocytes as a positive control (30x magnification) stained with JC-1. MitoPT JC-1 accumulates in negatively charged (normal) mitochondria and aggregates, fluorescing red upon excitation. When mitochondrial membrane potential is disturbed (abnormal), MitoPT is distributed in its monomeric form throughout the cell, fluorescing green upon excitation. (**F**) Ratio of red to green fluorescence signal intensities across 10 sections of individual oocytes (wild type n = 6, *mdr1a* mutant n = 3, positive control n = 10) in random fluorescence units (RFUs). Wild type oocytes exhibit higher red to green fluorescence ratios, indicating that the cells contain more mitochondria with normal negative membrane potential than mutant oocytes. Quantification performed using ImageJ.

In parallel with the increased sensitivity to cyclophosphamide, immature, unchallenged germinal vesicle stage oocytes of *mdr1a* mutant mice showed significantly increased oxidative stress at baseline relative to their wild type counterparts (Fig. 4A). Oxidative stress in the form of mitochondrial superoxide was detected by MitoSOX, an indicator used on live cells. The two cohorts of oocytes overall were significantly different from each other (Fig. 4B). Cellular oxidative stress was also observed via CM-H_2_DCFDA staining, an indicator that is oxidized by ROS in the cytoplasm (Fig. 4C). The two cohorts of oocytes were again significantly different from each other, and we also note that the fluorescence intensity was more variable in the *mdr1a* mutant population in both experiments (Fig. 4D).

### Mitochondrial membrane potential is disturbed in *mdr1a* mutant oocytes

Given the abnormal metabolite accumulations, increased ROS, and morphological changes in the mitochondria of the *mdr1a* mutant mice, we determined that we needed to perform an *in vivo* assay to test mitochondrial physiology. We utilized the JC-1 assay given that it had been performed in oocytes^28^ and could yield ratios indicating changes in mitochondrial homeostasis. JC-1 dye diffuses into cells and enters mitochondria with normal negative membrane potential. The dye then accumulates as aggregates in these healthy mitochondria and fluoresces red. If mitochondrial membrane potential is disturbed, JC-1 remains distributed in the cell as a monomer and fluoresces green. In this experiment, we observed that oocytes of *mdr1a* mutant mice had lower ratios of red to green fluorescence (p = 0.0089), very similar to levels detected in CCCP treated positive controls, which is indicative of disturbed, non-negative mitochondrial membrane potential and potentially apoptosis (Fig. 4E,F).

### MDR-1 localizes to the oocyte mitochondrial membrane

**Figure 5:**
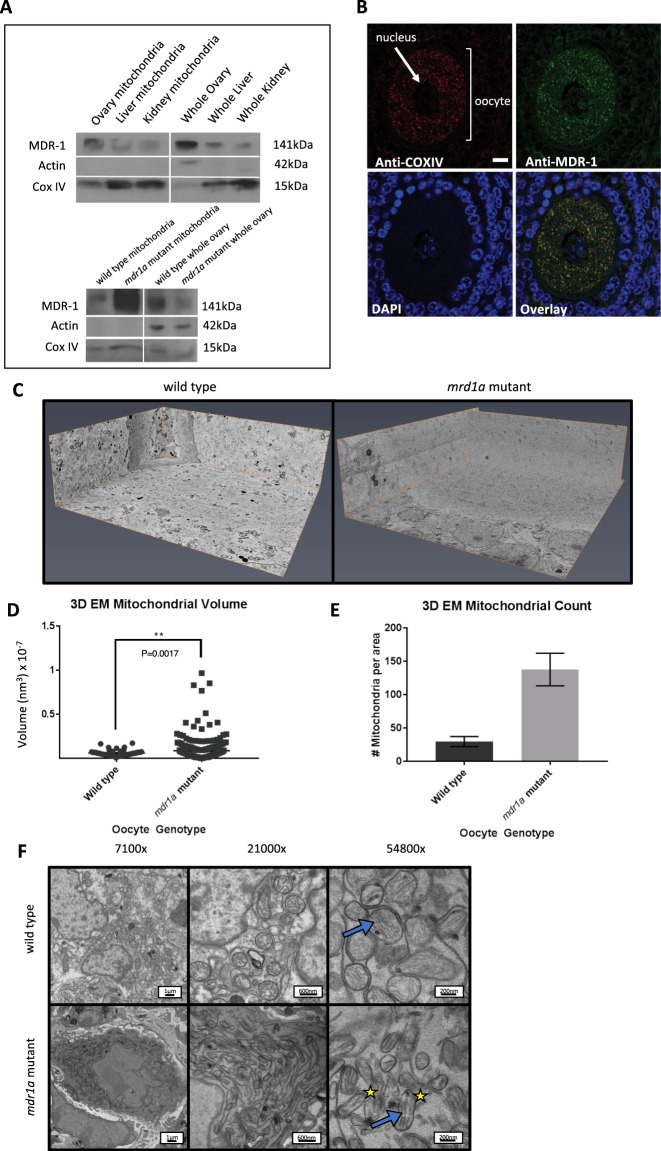
MDR-1 is present in oocyte mitochondria and mutants display abnormal mitochondrial morphology. (**A**) Western blot of MDR-1 protein in wild type whole organ and isolated mitochondrial samples, and a western blot of MDR-1 protein in wild type vs. *mdr1a* mutant ovarian mitochondrial isolates as well as whole ovaries. CoxIV was used as a control to confirm presence of mitochondria in each sample, and Actin was used as a cytoplasmic control to confirm a lack of contamination in the mitochondrial isolates. Blots were cropped for display in this figure. Full-length blots are presented in (Fig. S6B). (**B**) Immunofluorescence staining of the localizations of Cox IV and MDR-1 in an oocyte. The image shows an oocyte with a visible nucleus (blue DAPI staining), surrounded by granulosa cells (blue DAPI staining). Ovary sections of wild type mice were stained with anti-Cox IV (red), anti-MDR-1 (green) antibodies, and DAPI. Scale bars: 10 µm. (**C**) 3D electron microscopy images depicting 3D reconstructions of wild type and mutant oocytes. Dimension of 3D reconstructed volume: 40 um × 35 um × 12 um. (**D**) Mitochondrial volume, calculated from 3D reconstructed oocyte images. Wild type and mutants showed significant differences in oocyte mitochondrial volume. (**E**) Mitochondrial count, from 3D reconstructed oocyte images. There were more mitochondria present in mutant oocytes albeit not significantly. (**F**) Transmission electron microscopy images of ovarian mitochondria. Wild type ovarian mitochondria have more distinct and organized membranes and cristae when compared to *mdr1a* mutant mitochondria. Images were taken at 3 increasing magnifications. Blue arrows point to cristae, yellow stars indicate oblong mitochondria.

In an effort to further study the role of MDR-1 in oocyte mitochondrial function, we examined the localization of MDR-1 protein in wild type oocytes of fixed wild type ovaries. Intriguingly, we found that MDR-1 signal is distinctly present in a punctate perinuclear pattern in the cytoplasm of oocyte and its surrounding granulosa cells, and these puncta of MDR-1 signal overlap extensively with the signal of specific mitochondrial marker complex IV subunit (COX IV) (Fig. 5B). We also noticed that the overlap of MDR-1 and COX IV is not complete (Fig. 5B). Immunoblot was performed to test for concurrent expression in mitochondrial isolates. Mitochondrial isolates from wild type mouse ovaries, livers and kidneys prepared for immunoblot, and prepared for immunofluorescence on fixed whole ovaries were incubated with two different commercial antibodies made to separate regions of the MDR-1 protein showed concurrent expression in wild type oocyte and granulosa cell mitochondria (Fig. 5A). This experiment was repeated with wild type and *mdr1a* mutant ovarian mitochondrial isolates, as well as whole ovaries (Fig. 5A).

### Mitochondria are morphologically different when mice lack functional MDR-1

Metabolite analysis indicates significant differences in the mitochondria in *mdr1a* mutant ovaries. Therefore, we directly tested mitochondrial morphology and density in oocytes. The *mdr1a* mutant and wild type ovaries were fixed for 3-D scanning electron microscopy and histomorphometry was performed (Fig. 5C). The mitochondria of *mdr1a* mutant oocytes were larger, and more numerous than that in the wild type cells (Fig. 5D,E). Consistent with this, we persistently detect higher (though not statistically significant) copy number of mitochondrial DNA (mtDNA) in the mutant oocyte in comparison to that in the wild type ovary (Fig. S2). However, it is not clear if these morphological and numeral differences are causative to differences in metabolites and oxidative stress we observed or *mutatis mutandis*. Mitochondria in whole ovary sections were also observed with TEM, where *mdr1a* mutant ovarian mitochondria appeared to have fewer, more swollen cristae and have a more elongated morphology compared to wild type (Fig. 5F).

### *mdr1a* mutant animals produce more oocytes in normal estrus and when superovulated, but do not give birth to more pups

**Figure 6:**
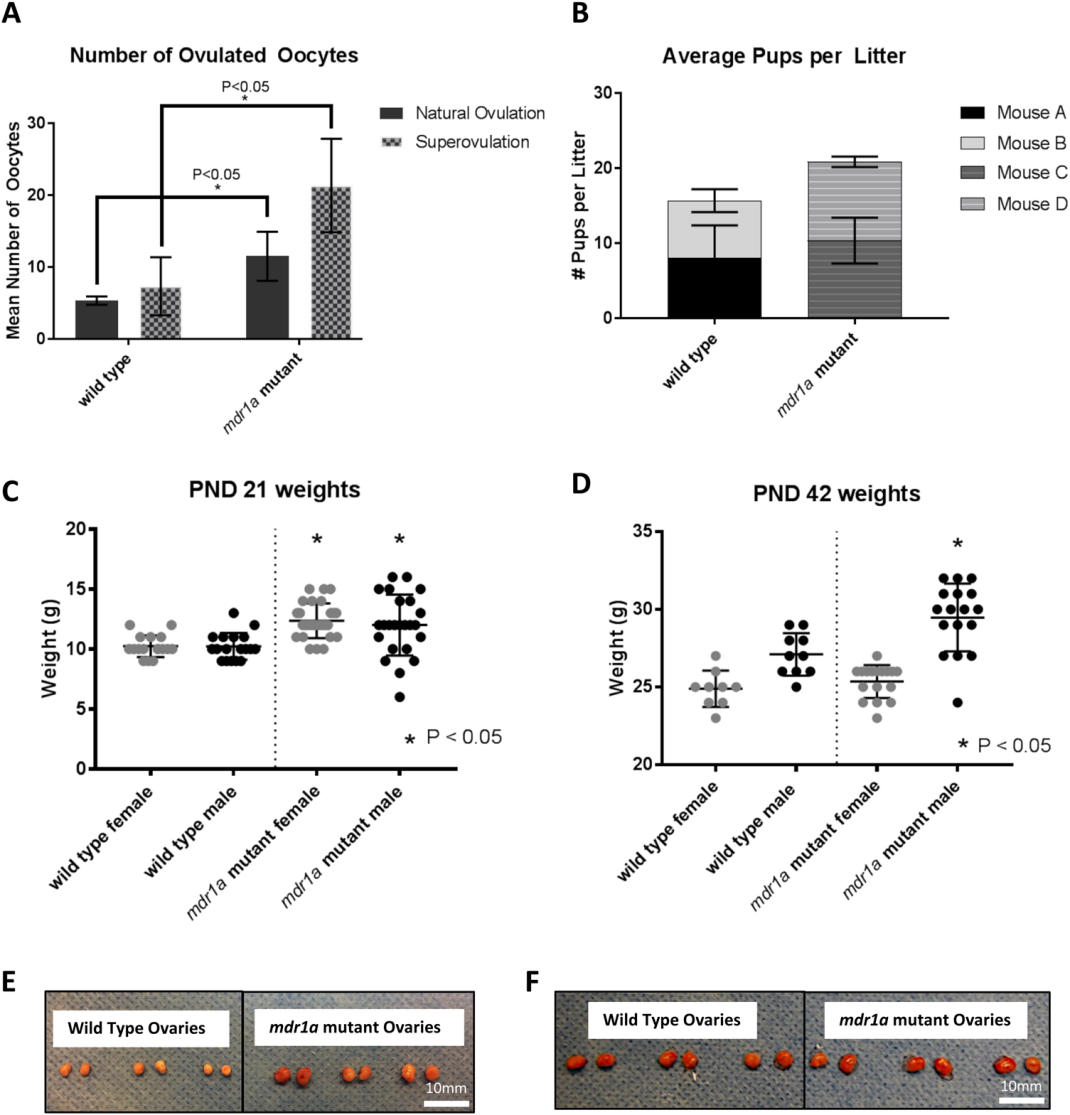
mdr1a mutant animals produce more oocytes at ovulation, but do not give birth to more pups. (**A**) *mdr1a* mutant mice produced statistically more oocytes at natural ovulation (n = 4, p = 0.0340), and upon superovulation (n = 3, p = 0.0436). (**B**) Each bar section represents one female mouse (Mouse A, B, C or D) that gave birth to three successive litters. Pup counts across these three litters were averaged for each mouse. The average number of pups born to the mice in each group weren’t statistically different. (**C**,**D**) *mdr1a* mutant male pups, born to mutant female and mutant male pairs, weigh more than control and mutant female pups. Pups were weighed at (**C**) weaning age (21 days) and then again at (**D**) 6 weeks (42 days). (**E**) Ovaries of virgin 2 month *mdr1a* mutant mice were significantly larger than ovaries of virgin wild type mice of exactly the same age. (**F**) Ovaries of virgin superovulated mice of the same age exhibited no difference in size.

In this experiment, we endeavored to characterize oocyte quality in mutant mice given the potential for mitochondrial compromise. We sacrificed mice in natural estrus, and superovulated other animals with pregnant mare serum gonadotropin (PMSG) and collected oocytes from the oviducts after sacrificing the mice. The oocytes were counted directly upon release from the oviduct. Strikingly, mutant animals ovulated significantly more oocytes (p = < 0.05) than wild type when stimulated with PMSG and triggered with human chorionic gonadotropin (hCG), suggesting that the *mdr1a* mutant ovary ovulates higher number of oocytes than the wild type ovary does (Fig. 6A). We repeated these experiments with mice who ovulated naturally in estrus. Mutants ovulated significantly more oocytes compared to wild type (p = < 0.05). Anecdotally, we observed increased oocyte lysis in the *mdr1a* mutants when removing the cumulus during pipetting (data not shown). However, we found no differences between the morphology of the oocytes from either group. Intriguingly, we found that in an unstimulated state, the ovaries from *mdr1a* mutant mice are significantly larger in size compared to those from wild type mice (Fig. 6E). However, the ovarian size difference is lost when mutant and wild type mice are superovulated with PMSG and hCG (Fig. 6F). However, the total number of follicles present in the unstimulated wild type and mutant ovary are not significantly different (Fig. S3). We did not find differences in the proportion of follicles at each stage in the ovaries of the two genotypes. We also examined the level of steroid hormone estradiol and markers for ovarian reserve, including inhibin B, estradiol (E2), and Anti-Mullerian Hormone (AMH). No difference in the level of these markers was present between the wild type and mutant in the ovary or the serum (Fig. S4). We then evaluated whether the increased oocyte production in *mdr1a* mutant mice led to increased fertility. Curiously, the litter sizes of mutant and wild type females are approximately the same (Fig. 6B).

### The influence of *mdr1a* mutation on the general health of the animals

We then evaluated the impact of MDR-1 dysfunction on the development and health of offspring. At postnatal day (PND) 21, weaning age, female and male *mdr1a* mutant mice weighed significantly more than female and male progeny of wild type mice (p < 0.05) (Fig. 6C). However, at PND 42, that significance disappeared for the female mice while *mdr1a* mutant males remained significantly heavier than wild type males (p < 0.05) (Fig. 6D). The ratio of female to male mice in the *mdr1a* mutant colony was also higher than the female/male ratio in the wild type colony, though this difference was also not significant (Fig. S5).

Over one year of breeding, 6 *mdr1a* mutant mice developed tumors as diagnosed by Brown University’s Animal Care Facility attending veterinarian and technicians and 4 out of 6 cases were confirmed by pathologic diagnosis to be lymphomas and myoepitheliomas (Fig. S5). This incidence was calculated to be a rate of 2.12% occurrence in the total mouse colony, and 2.73% in females. Though no instances of any tumors occurred in the wild type colony over one year of breeding, the difference in prevalence between colonies was determined not to be significant.

## Discussion

Our findings demonstrate that MDR-1 is expressed in the oocyte mitochondrion. The lack of complete colocalization of MDR-1 and COXIV, a mitochondrial marker, suggests that MDR-1 protein is likely present on some, but not all mitochondrial membranes in the oocyte. This could indicate a variation in normal wild type mitochondrial physiology. We also show that functional MDR-1 is essential for several critical and quantitative physiological functions in the maintenance of normal mitochondrial physiology, such as detoxification, mitochondrial homeostasis, and metabolite exchange in the ovary and oocyte. Remarkably though, we learned that transcription, or at least steady state mRNA accumulation, also relies in some way on MDR-1 functionality. Though MDRs are sentinel transporters at the plasma membrane, the discovery of their expression in the mitochondrion broadens our perspective of its roles in germ cell mitochondrial homeostasis. It would be logical to conclude then that dysfunctional MDR-1 in the germ line and/or in the soma of the ovary would, over time, lead to organelle and cellular changes that could lead to abnormal female gametes, ovarian dysfunction, and metabolic alterations in embryos or offspring.

Our data lead us to conclude that MDR-1 appears to foster normal oxidative phosphorylation; many of the polar metabolites found overexpressed in this study are important products and or substrates in the TCA cycle (Fig. 2B). These metabolites may be substrates of MDR-1, and in the absence of the transporter, they then over accumulate in the cell. Another possibility is that MDR-1 is essential for mitochondrial homeostasis, and without it the cell has an increase in steady state levels of these metabolites associated with oxidative stress. Alternatively, these values may come from mis-regulation of various steps within the metabolic pathway resulting directly or indirectly from the absence of this one MDR. Currently we cannot distinguish between these various possibilities, but it is important to emphasize that these metabolic values are from untreated cohorts and reflect steady state levels of the metabolites. We hypothesize that over time in these individuals, such differences in metabolism/metabolites could play into multiple secondary effects in physiology and that over the reproductive lifetime of a human lacking normal MDR-1 function, such differences may alter oocyte viability and quality, compromising normal reproduction. Specifically, cytidine and xanthosine specifically are involved in purine metabolism and pyrimidine biosynthesis, which could indicate cellular stress^25^. 3-phosphoglycerate consumption is very intriguing in that it is an intermediate of glycolysis which may further suggest that *mdr1a* mutants preferentially use aerobic glycolysis. Additionally, Zymosterol is a precursor to cholesterol in the steroid pathway. The long-term impact of this difference is still unclear, but since steroidogenesis occurs in the mitochondrion, this could signal a subclinical hormonal production deficit that we currently cannot detect in our mouse colony.

Our findings also suggest that *mdr1a* mutant oocytes have morphologically abnormal mitochondria (Fig. 5), increased ROS formation and abnormal mitochondrial membrane potential (Fig. 4). This dramatic difference in oocyte physiology may reveal an ultrasensitive pathway intersect with the mitochondrion, MDR-1, and the oocyte. The measurement of the exact cellular respiration of oocytes is challenging, despite the egg having the most mitochondria of all the cells, because they are the rarest cells in the body,  making oxygen consumption and extracellular acidification challenging to study in their short window of viability. However, our results refocus our interest on the relationship between MDRs, mitochondria, and mRNA levels in cells of the ovary. In support of this concept are results from multidrug resistant hepatocellular cancer cell lines that overexpress MDR-1. These cells show that the transporter is in fact present in the membrane of mitochondria^18^ and may contribute to the phenotype reported here of abnormal metabolite accumulation. We documented here that *mdr1a* mutation caused over-accumulation of key components of the TCA cycle as well as possible perturbation of the mitochondrial physiology. While some of the over accumulated metabolites in the mouse may be innocuous and the mitochondrial phenotype may not be severe, these disturbances may trigger secondary reactions and, in the least, reflect abnormal feedback on essential metabolic pathways.

These metabolic disturbances associated with lack of functional MDR-1 may impact unknown processes further cementing a link between MDR-1 and mitochondrial bioenergetics. The impact of altered metabolism may significantly impact reproduction and normal embryogenesis given the reliance on the maternal contribution of mitochondria. Aberrant metabolism may explain the abnormal ovarian size of the mutants and their hyperstimulation response to both endogenous and exogenous gonadotropins yielding high numbers of poor quality oocytes (Fig. 6), as it appears that the *mdr1a* mutant ovaries may be in a constantly stimulated state. Another viable theory is that the loss of functional MDR-1 may lead to increased follicle recruitment incrementally as they age to compensate for poorer oocyte quality. And while our data from 6-week mice suggest that the mutant ovary exhibits normal capability of steroidogenesis and normal ovarian reserve at young ages, we hypothesize that increased recruitment of follicles over time may more rapidly deplete the ovarian reserve and lead to a diminished ovarian reserve at older ages. In future, we plan to examine aged *mdr1a* mutant mice to determine if there are more obvious follicular and hormonal differences. Our observation of increased oocytes not yielding additional pups is likely related to the oocytes themselves and less likely to be related to the uterine environment. However, we plan to test that hypothesis by evaluating embryogenesis after *in vitro* fertilization and transferring *mdr1a* mutant embryos to wild type pseudopregnant mice  to observe their fetal development with ultrasonography. Subsequently, we will observe the uterine horns of the recipient mice to determine if there are increased embryonic resorptions. This may also help us resolve the observations of increased pup size at PND 21 of the *mdr1a* mutants which is retained in the male pups at PND 42. The increased pup size may signify a type of “twin-twin transfusion” or perhaps a metabolic syndrome-like process which merits further investigation and long-term follow up (Fig. 6C,D). While we did not find a significant difference, sex ratios could also be altered as we did learn that *mdr1a* mutants had a female-to-male ratio of 110:79, while wild type animals had an almost equal female-to-male ratio of 80:78 (Fig. S5).

Consistent with MDR-1 as an ovarian sentinel transporter, Ho *et al*. found that the gastrointestinal tract of *mdr1a* knockout mice had patterns consistent with inflammatory bowel disease, which is a well-known risk factor for the development of colon cancer^11^. Epidemiologic data suggest also that women who possess mutations in MDR-1 (P-glycoprotein) *C3435T* mutations have an increased risk of breast cancer^29^. This highlights the role of MDRs as protectors from chronic inflammation, cancer, as well as infertility. The infertility and perhaps accelerated aging due to oxidative stress may be a harbinger on the continuum of early organ dysfunction ultimately leading to ovarian cancer. While we do not report any ovarian tumors in our colony, one year of breeding data shows a possible impact of the loss of MDR-1 on development of tumors, specifically lymphomas and myoepitheliomas. This phenomenon, not observed in the wild type, lends support of the theory that loss of MDR-1 is potentially associated with a pro-cancer phenotype.

Loss of ABCB7, another family member of MDR transporters, caused increased oxidative stress in *C. elegans*^30^, also seen in the present study in a mammalian oocyte. While this is a whole animal germline mutation, there appear to be dramatic changes in the ovary autonomously which may indicate decreased mitochondrial activity which is associated with tumorigenicity due to enhanced ROS production. We also observed increased formation of ROS in the oocytes of the *mdr1a* mutant mice. Wallace *et al*.^31^ showed that nuclear hydrogen peroxide increased the transcription of selected genes that favor tumor progression. Specifically, the over-accumulation of pyruvate^32^ and succinate^33^, which we observed in the *mdr1a* mutant ovaries, has been reported to stabilize HIF1α. The ability of metabolites to modify HIF1α leads to a shunting of cells to glycolysis which is the preferred, quicker metabolic pathway for tumors to produce ATP. HIF1α also induced MDR expression in the cell lines T84 and Caco-2 as well as non-transformed human epithelial cells^34^. Our results, combined with tangential work in the field, suggest that the roles of MDRs, mitochondria, gene expression, and oocyte dysfunction are highly interconnected and may reveal an underlying mechanistic explanation for patients seen in the infertility clinic who may face an increased risk for tumorigenesis and may require increased surveillance.

Based on the data obtained, we plan to further investigate the basis for poor oocyte quality in the *mdr1a* mutants. We plan to record meiotic spindle assembly in MII oocytes since the microtubules rely on ATP to assemble and form a polar body. We also plan to evaluate how overexpression of* mdr1a* mRNA and antioxidant treatment of mutant oocytes may facilitate normal mitochondrial physiology and give rise to embryos that are developmentally competent. The inability of increased number of *mdr1a* mutant MII oocytes to produce larger litter sizes, along with the difference we observed in sex ratios between wild type and mutant colonies, suggests that loss of MDR-1 manifests not only in metabolic and mitochondrial changes within the oocyte, but also in long term effects on reproduction. Another future direction of this study would be to investigate human *in vitro* fertilization (IVF) patient *ABCB1* SNPs and their clinical reproductive and neonatal outcomes and then subsequently study how clinically relevant SNPs *in vitro* effect on mitochondria in a cell line which may have certain advantage over oocytes.

In this study we documented that MDR-1 is expressed on the mitochondrional membrane in the oocyte. We also showed that MDR-1 and the mitochondrion work cooperatively to protect and regulate mitochondrial homeostasis. Our findings shed light on the normal physiology of this essential transporter in the oocyte which may have broad implications for reproduction and long-term health outcomes.

## Methods

### Animals

An animal protocol was obtained from the Institutional Animal Care and Use Committee (IACUC) Protocol numbers 1407000080 and 1710000312. Wild type (CF-1) and P-glycoprotein (*mdr1a*) mutant mice were obtained from Charles River Laboratories, Wilmington, MA. Mice were sacrificed by CO_2_ asphyxiation and cervical dislocation. All experimental protocols were approved by the IACUC at Brown University, and all methods were carried out in accordance with relevant guidelines and regulations.

### Genotype characterization

To examine the presence of exon 23 in the *mdr1a* transcript, RNA was extracted from whole ovaries from 6-week wild type and *mdr1a* mutant mice using RNeasy RNA extraction Kit (Qiagen Inc., Germantown, MD). Reverse transcription was performed using Maxima First Strand cDNA Synthesis Kit (Thermo Fisher Scientific, Waltham, MA). Primers F1 and R1 were used for the amplification of the region surrounding *mdr1a* exon 23 (Supplemental Table 1). DNA was extracted from tail snips of wild type and *mdr1a* mutant mice using DNeasy DNA extraction kit (Qiagen Inc., Germantown, MD). Primers F2 and R2 were used for the amplification of the viral LTR sequence and *mdr1a* exon 23 (Supplemental Table 1). All amplicons were extracted from the gel using Purelink gel purification kit (Thermo Fisher Scientific, Waltham, MA), and sequenced by Genewiz sequencing service (South Plainfield, NJ).

### Protein domain and structure analysis

The peptide sequence of MDR-1 was analyzed using online tool ProSite (https://prosite.expasy.org/). The 3D structure of MDR-1 protein was predicted by PyMol according to the RefSeq wild type MDR-1 peptide sequence.

### Efflux assay with calcein-AM

Wild type and *mdr1a* mutant GV oocytes were isolated via mechanical extraction and experiments were completed as reported in Brayboy *et al*.^25^ were recorded using EVOS FL Auto Cell Imaging System (ThermoFisher Life Technologies) using the time lapse feature equipped with a 37 °C degree temperature stage and Trigas chamber (90% Nitrogen, 5% CO2 and 5% O2 mimicking IVF-incubator conditions). To create a positive control, wild type oocytes were incubated with 25 mM PSC 833, a specific inhibitor for MDR1a, for 15 minutes before Calcein-AM incubation. Images were quantified over time using ImageJ.

### Cyclophosphamide treatment of *mdr1a* mutant and wild type mice

Adult (6 week) female mice were injected intraperitoneally with saline, 75 mg/kg, or 150 mg/kg cyclophosphamide and then sacrificed at 24 or 48 h following injection. Ovaries were imaged as described in Brayboy *et al*.^16^.

### Metabolomic profiling

Whole mouse ovaries from wild type (n = 3) and *mdr1a* mutants (n = 3) were dissected from 6-week adults. They were snap frozen in liquid nitrogen and stored at −80 °C. Metabolic analyses were performed at Massachusetts Institute of Technology Metabolite Profiling Facility, Whitehead Institute for Biomedical Research, Cambridge, MA. Each tissue (~10 mg) was homogenized on dry ice in 600 ml methanol using a disposable Pellet Pestle (Kimble-Chase, Rockwood, TN). After addition of 300 ml water and 400 ml chloroform (HPLC grade), samples were vigorously shaken for 10 min at 4 °C, then centrifuged at 15,000xg at 4 °C. The two phase-separated layers were collected separately, dried by Speedvac, and stored at −80 °C. For polar metabolite analysis, samples were resuspended in 100 ml water, and 1 ml was injected for LC/MS analysis. Lipid analysis was performed on the same samples following resuspension in 50 ml of 65/30/5 acetonitrile:isopropanol:water (v/v/v), and 5 ml was injected for LC/MS analysis.

### *mdr1a* mutant transcriptome

Whole ovaries (n = 6 wild type and n = 6 *mdr1a* mutant) were dissected from 6-week mice and snap-frozen in liquid nitrogen, stored at −80 °C, and sent to Genewiz, South Plainfield, NJ, for transcriptomic sequencing. Analysis was performed by the Brown University biostatistics core. The ovaries were rRNA depleted so that coding and noncoding mRNA could be evaluated. Libraries were prepared using standard techniques and reagents and 2 × 150 paired reads were obtained from an Illumina platform. Identified genes were then analyzed for differential gene expression between the two groups, and further analyzed using the Ingenuity Pathway Analysis (Qiagen). The transcriptomic sequencing results were validated by analyzing the expression of top differentially expressed protein-coding genes (ranked by adjusted p-value) with greater than 2-fold expression differences (*acsf2*, *c1rb*, *mid1*, *zbtb8b*, *gulo*, *tspan11*) in 6–7 month wild type and *mdr1a* mutant mice using quantitative PCR (qPCR). The primers used for the qPCR are listed in Supplemental Table 1.

### Natural ovulation and superovulation for metaphase II (MII) oocyte isolation

For superovulation, wild type and *mdr1a* mutant mice (8 weeks) were injected intraperitoneally with 5 IU pregnant mare serum gonadotropin (PMSG, Bioworld, Dublin, OH). 48 hours later, mice were injected with 5 IU human chorionic gonadotropin (hCG, Sigma-Aldrich, St. Louis, MO). Mice were euthanized 13–14 hours after hCG injection. For natural ovulation, vaginal lavage was performed on mice with 10 µL of milli q water in order to identify their stage of the estrous cycle. 10 µL of 0.2% methylene blue in 50% methanol was added to the sample, and total volume was placed on a slide with a glass coverslip. Slides were viewed on the EVOS FL Auto Cell Imaging System (ThermoFisher Life Technologies), and mice were determined to be in estrous if they exhibited cornified squamous epithelial cells only, with no nucleated epithelial cells or leukocytes. Mice in estrous were sacrificed. In both conditions, ovariectomies were performed and MII oocytes were isolated from the ampulla of the oviduct and counted.

### Mitochondrial superoxide detection in oocytes

Germinal vesicle (GV) oocytes were mechanically extracted from 6-week *mdr1a* mutant and wild type mouse ovaries. Oocytes were stained in MitoSO Red Mitochondrial Superoxide Indicator (Invitrogen M36008, Eugene, OR) for 20 min as described by Brayboy *et al*.^16^. Oocytes were then co-stained with MitoTracker Green (Molecular Probes M-7514, Eugene, OR) for 10 minutes to allow for visualization of colocalization. Some oocytes were incubated in 20 mM H_2_O_2_ for 5 minutes prior to MitoSOX/MitoTracker Green staining as a positive control. Oocytes were imaged on an Olympus FV3000 Confocal Microscope and ImageJ was used to quantify fluorescent intensity.

### Cellular superoxide detection in oocytes

Germinal vesicle (GV) oocytes were mechanically extracted from 6-week *mdr1a* mutant and wild type mouse ovaries. Oocytes were imaged prior to incubation as a baseline negative control. Some wild type oocytes were then incubated in 20 mM H_2_O_2_ for 5 minutes as a positive control (n = 5). Oocytes were stained with 10 µM CM-H_2_DCFDA (Invitrogen C6827), Eugene, OR) for 30 minutes (n = 5 wild type, n = 9 mutant), and imaged with a confocal laser scanning microscope (upright Zeiss LSM800, Carl Zeiss Inc, Thornwood, NY). ImageJ was used to quantify fluorescent intensity.

### Mitochondrial membrane potential (JC-1)

MII oocytes were isolated from superovulated 8-week wild type and *mdr1a* mutant mice. The MitoPT JC-1 Assay Kit (ImmunoChemistry Technologies, Bloomington, MN) was used. 50 µM carbonyl cyanide 3-chlorophenylhydrazone (CCCP) incubations at 37 °C for 1 hour were used as a positive control. Oocytes were incubated in either 1X MitoPT reagent, or 1X Assay Buffer as a negative control, for 30 min at 37 °C, protected from light. Oocytes were transferred to clean drops of 1X Assay Buffer in a 35 mm Fluorodish. Drops were covered in mineral oil and imaged immediately on an Olympus FV3000 Confocal Microscope. Ratios of red to green fluorescence in ten sections of each individual oocyte (wild type n = 6 and *mdr1a* mutant n = 3) were calculated using ImageJ. Ratios of red to green fluorescence intensity were averaged for each oocyte and significance was analyzed by unpaired t-test.

### Mitochondria isolation

Whole ovaries, livers, and kidneys were dissected from 6-week wild type and *mdr1a* mutant mice. Organs were homogenized in 10 mL/g mitochondrial homogenization buffer (5 mM Hepes, 250 mM Sucrose, 1 mM EDTA, 25 mM NaF, 1 mM NaVanadate, with complete mini EDTA-free protease inhibitor cocktail tablets and 1 µM microcystin). Supernatant was centrifuged at 500xg for 10 min at 4 °C, and resulting supernatant was centrifuged again at 3000 × g for 10 min at 4 °C. Supernatant was discarded, and the pellet was washed with 2 mL homogenization buffer and centrifuged at 3000xg for 10 min at 4 °C. Supernatant was discarded and pellet was resuspended in 600 µL extraction buffer (10 mM Tris-Base, 5 mM EDTA, 50 mM NaCl, 30 mM Sodium Pyrophosphate, 50 mM NaF, 100 µM NaVanadate, 1% Triton X-100, with protease inhibitor tablet). Samples were centrifuged at 16,000 rpm for 15 min at 4 °C, and supernatant was added to equal volume 2X sample buffer and stored at −20 °C.

### Western blot

Whole ovaries, livers, and kidneys and mitochondrial isolates were run on a western blot and incubated with mouse monoclonal anti-COX IV (abcam ab14744, 1:1000), mouse monoclonal anti-Actin (abcam ab8226, 1:1000), and rabbit monoclonal anti-P-gp (abcam ab170904, 1:1000) primary antibodies in 2% BSA overnight at 4 °C. Blots were labeled with corresponding rabbit anti-mouse or goat anti-rabbit HRP-conjugated secondaries, (1:3000) for 1 hour. HRP-labeled proteins were developed in ECL Western Blotting Substrate (Thermo Fisher Scientific, Waltham, MA).

### Immunofluorescence

Wild type ovaries (6 weeks) were fixed in 2.5% glutaraldehyde, 2% paraformaldehyde, 0.15 M sodium cacodylate buffer, and 0.1 M sucrose. Ovaries were embedded in paraffin, sectioned at 7 µm, and mounted on slides. Slides were rehydrated in 100% xylene, graded ethanols, washed in PBS, 0.01 M sodium citrate at 95 °C for 20 min, and room temperature sodium citrate for 20 min. Slides were washed 2x in ddH_2_O for 2 min and 3x in PBS for 3 min, and blocked in incubation buffer (1% BSA, 10% fetal calf serum, 0.1% Tween-20 in PBS) for 1 hour at room temperature. Slides were then incubated in 0.1% Sudan Black B (Sigma Aldrich, St. Louis, MO) in 70% ethanol for 20 min, washed 3x in PBS for 5 min, and jet washed with PBS. Primary antibodies mouse monoclonal anti-COX IV (abcam ab14744, 1:200) and goat polyclonal anti-P-gp (Santa Cruz sc-1517, 1:200) overnight at 4 °C in a humidity chamber, and secondary antibodies fluorescent red anti-mouse (1:200) and green anti-goat (1:200) for 1 hour at room temperature were used. Samples were washed and mounted with Vectashield Antifade Mounting Medium with DAPI (Vector Laboratories, Burlingame, CA) and imaged with a confocal laser scanning microscope (upright Zeiss LSM800, Carl Zeiss Inc, Thornwood, NY).

### Electron microscopy

Ovaries from 6-week wild type and *mdr1a* mutant mice were fixed in 2% paraformaldehyde and 2.5% glutaraldehyde with 0.15 M sodium cacodylate buffer and 0.1 M sucrose. Samples were washed in 0.15 M sodium cacodylate buffer with 2 nM calcium chloride. They were post fixed with 2% osmium tetroxide/potassium ferrocyanide mixture, 1% thiocarbohydrazide, then 2% osmium tetroxide with appropriate rinses between staining steps. Specimens were en bloc stained with 1% uranyl acetate overnight and Walton’s lead aspartate pH 5.5 at 60 °C for 30 min. Samples were dehydrated with graded ethanols, and infiltrated with propylene oxide twice, 20 min each.

For transmission electron microscopy, samples were infiltrated with Araldite-EMbed embedding medium/propylene oxide 1:2, 1:1, and 2:1 on a rotator 2 hours to overnight, followed by 100% embedding medium overnight. Samples were rotated with fresh embedding medium for at least 2 hours, embedded in beem capsules, then cured at 60 °C for 24 hours.

For 3-D electron microscopy, samples were infiltrated with Araldite Embed 812 embedding medium (Electron Microscopy Science, Hatfield, PA), and embedded and cured at 60 °C. Blocks were imaged using an Apre VS in Volumescope mode (ThermoFisher Scientific, Hillsboro, OR) at 3.0 KV accelerating voltage, with water vapor at 40 Pa, and using the dedicated VS DBS detector with a 100pA probe current and 3 µs dwell time. A large overview region was collected with a 400 µm HFW and 130 µm pixels. Two or three ROIs were used for each sample, and all were collected with 10 nm pixel size and 40 µm HFW. 300 serial sections were cut for each block at 40 nm each for a total volume of 40 µm × 40 µm × 12 µm. Amira 3D Image analysis was performed using greatest mitochondrial diameter, every 200 slices of 40 nanometers each. Mitochondrial volume was calculated using measured circumference.

### Statistics

RNA sequencing counts were used for gene differential expression analysis. Genes with at least 1 count per million in at least 2 samples were included. Expression data were normalized with weighted trimmed mean of M-values (TMM). “edgeR” R package was used to identify differentially expressed genes between mdr1a mutant and wild type samples. Genes with FDR < 0.05 were considered significant.

Gene ontology (GO) analysis was performed on the expression profile from RNA sequencing of *mdr1a* mutant vs. wild type cells using conditional hypergeometric test in “GOstats” R package with org.Mm.eg.db annotation package. Genes identified as differentially expressed were used in GO term enrichment. A Bonferroni corrected p-value cutoff, 1.1e-5, was used to identify interesting GO terms. Terms with size less than or equal to 5 genes were omitted from the result as there is not enough power for the test.

All other data were analyzed by unpaired t-tests in GraphPad Prism.

## Supplementary information

Supplement

## Data Availability

The datasets generated during and/or analyzed during the current study are available from the corresponding author on reasonable request.
