# Supplementary material for: Dysfunctional MDR-1 disrupts mitochondrial homeostasis in the oocyte and ovary
Source: Sci Rep. 2019 Jul 3;9:9616. doi: 10.1038/s41598-019-46025-x (PMC6610133; doi:10.1038/s41598-019-46025-x)
Supplement: Supplementary file 1 — Supplement [file 41598_2019_46025_MOESM1_ESM.pdf]

Title: Dysfunctional MDR-1 disrupts mitochondrial homeostasis in the oocyte and ovary.

Haley Clark[a], Laura O. Knapik[a], Zijing Zhang[a], Xiaotian Wu[d], Mandar T. Naik[e], Nathalie Oulhen[c], Gary M. Wessel[c], Lynae M. Brayboy[abc*]
a Department of Obstetrics and Gynecology, Division of Reproductive Endocrinology and Infertility, Women & Infants Hospital of Rhode Island, Alpert Medical School of Brown University, 101 Dudley Street, Providence, RI 02905, USA
b Alpert Medical School of Brown University, 222 Richmond Street Providence, RI 02903, USA
c Department of Molecular Biology, Cell Biology and Biochemistry, Brown University 185 Meeting Street, Providence, RI 02912, USA
d School of Public Health Brown University, 121 South Main Street, Providence, RI 02903, USA
e Brown University Structural Biology Core, 70 Ship Street, Providence, RI 02903, USA

Article Type: Reproductive Biology
*Corresponding Author: Lynae Brayboy [Lynae_Brayboy@brown.edu]
Corresponding Author's Institutions: Women & Infants Hospital of Rhode Island and Brown University

Supplemental Methods

S.1 Ovarian Histomorphometry

Whole ovaries from 6-week old wild type and *mdr1a-/-* mice were embedded in paraffin, serially sectioned at 5 μm, and stained with hematoxylin and eosin. Every tenth section was imaged using an EVOS® FL Auto Cell Imaging System at 40X, and follicles were counted and staged using a method described by Flaws et al 1994 [16]. The number of corpora lutea were also quantified in each ovary.

S.2 Hormonal Assays

Wild type (n=6 animals) and *mdr1a-/-* mutant (n=6 animals) mice, 6 weeks old, in metestrus or diestrus confirmed by vaginal lavage were sacrificed for serum collection. The selected mice were exsanguinated, whole blood was allowed to clot at room temperature, the clot was disrupted, and the serum was centrifuged at 2000 g for 15 minutes and extracted. Serum was analyzed at the University of Virginia School of Medicine Center for Research in Reproduction Ligand Assay and Analysis core for quantitation of anti-Müllerian hormone (AMH), Inhibin B, follicle stimulating hormone (FSH), and estradiol. Whole ovaries were dissected and placed in PBS (phosphate buffer solution), disrupted with mortar and pestle, and sonicated to lyse. The homogenate was centrifuged at 2000 g for 5 minutes to remove supernatant. The homogenate was quantitated for AMH, Inhibin B, and estradiol.

S.3 Quantification of mitochondrial DNA (mtDNA)

To examine the mitochondrial DNA copy number in the ovarian tissue, DNA was extracted from whole ovaries of 3-month old wild type and *mdr1a-/-* mice. 1:1000 dilution of the ovarian DNA extraction was used as a template in subsequent qPCR analysis. Primers used for the amplification of mtDNA (16S and ND1) and nuclear DNA (b-actin) (Supplemental Table 1). For each genotype, ovary DNA extraction from 3 individual mice were examined. For each ovary, 3 technical replicates were processed, and the average cycle threshold (Ct) value of the three technical replicates were used for further calculations. The fold differences of the mtDNA-specific amplicons (*16S* and *ND1*) over nDNA-specific amplicons (actin) were derived from delta Ct (dCt) value, which was calculated as the difference between the average Ct of actin and mtDNA-specific amplicons.

Supplemental Figure Legends

Figure S1: qPCR validation of RNAseq results.
The expression of top protein-coding genes that are differentially expressed between wild type and *mrd1a-/-* (ranked by p-value) with greater than 2-fold expression differences were examined with qPCR. The qPCR was performed with 1:500 dilution of ovarian cDNA prep of wild type and *mrd1a-/-* mice. The cycle threshold (Ct) value for each gene was normalized to that of β-actin control. The expression foldchange of each gene was calculated basing on Ct. The expression foldchange of genes in *mdr1a-/-* were compared to that in wild type (set as 1) and the significance was analyzed using t-test.

Figure S2: Ovaries from mdr1a -/- mutant mice show a trend of higher mtDNA to nDNA ratio compare to that in ovaries from wild type mice.
The abundance of mitochondrial DNA (mtDNA) relative to nucleus DNA (nDNA) was inferred as the relative abundance of mitochondria-specific gene 16S and ND1 to nDNA-specific gene β-actin. For each genotype, ovaries harvested from 3 different mice were assayed as biological replicates. For each biological replicate, 3 technical replicates were included. The average cycle threshold (Ct) value of each gene from the 3 technical replicates was used to calculate the ΔCt, which was used to infer the abundance difference between the mitochondria-specific genes and actin. ΔCt (mt gene) = Ct(mt gene) – Ct(actin).

Figure S3: mdr1a-/- ovaries have similar follicle counts and proportions to wild type
No significant difference between mean total follicle per mouse of P-gp (n=3) vs wild type (n=3). There was not a significant difference amongst follicle proportions. 7 µm sectioned ovaries stained with hematoxylin and eosin imaged using an EVOS® FL Auto Cell Imaging System at 40X. Follicles categorized as: primordial, primary, preantral, antral and corpora lutea.

Figure S4: P-gp mutant (mdr1a-/-) ovaries have normal sex steroidogenesis and markers of ovarian reserve
Steroid assays were performed on both the sera and ovaries in the two groups. There were no differences detected.

Figure S5: Rate of tumor occurrence in wild type vs. mdr1a-/- mice.
Animal counts were recorded for one year of breeding at weaning age. 1 male case and 1 female case were confirmed by pathologic diagnosis to be lymphomas. 1 female case was confirmed to be a myoepithelioma. The female/male ratio in the *mdr1a-/-* mice was higher than that of wild type mice, though the difference was not significant.

Figure S6: Full-length western blots
(A) Western blot exhibiting expression of MDR-1 compared to control αTubulin. Cropped bands displayed in (**Figure 1A**) are shown in red boxes. (B) Western blot exhibiting expression of MDR-1 in ovarian mitochondria. Cropped bands displayed in (**Figure 5A**) are shown in red boxes. (C) PCR amplification of cDNA template. Cropped bands displayed in (**Figure 1C**) are shown in red boxes. (D) PCR amplification of genomic DNA template. Cropped bands displayed in (**Figure 1C**) are shown in red boxes.

Table S1: List of primers
Primers used for PCR and qPCR.

Figure S1

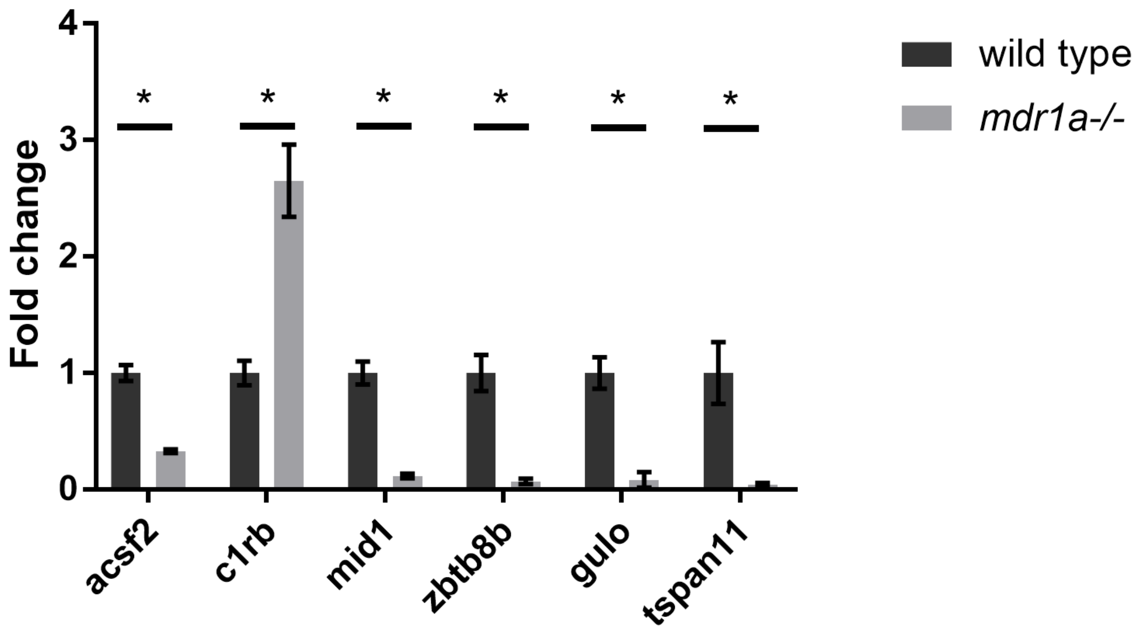

Fold change
4
3
2
1
0
*
*
*
*
*
*
acsf2
c1rb
mid1
zbtb8b
gulo
tspan11
wild type
mdr1a-/-

Figure S2

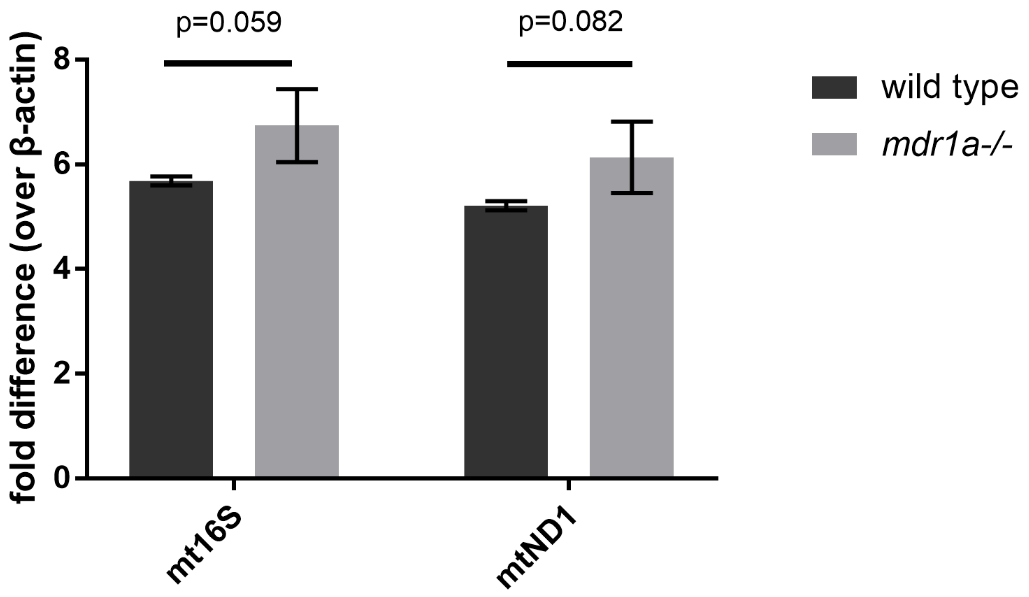

p=0.059
p=0.082
fold difference (over β-actin)
8
6
4
2
0
mt16S
mtND1
wild type
mdr1a-/-

Figure S3

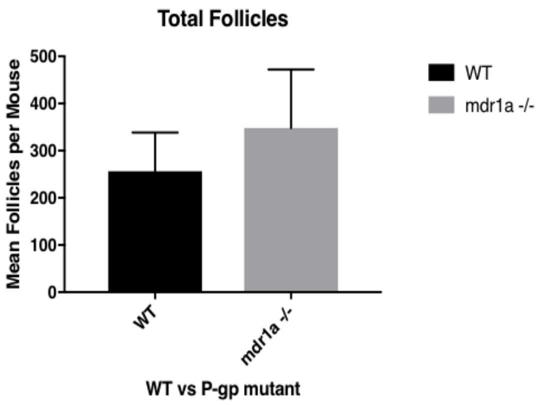
Total Follicles
Mean Follicles per Mouse
500
400
300
200
100
0
WT
mdr1a -/-
WT vs P-gp mutant
WT
mdr1a -/-

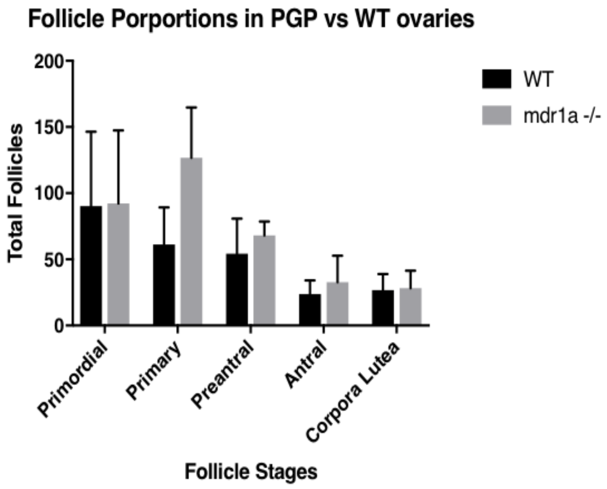
Follicle Porportions in PGP vs WT ovaries
Total Follicles
200
150
100
50
0
Primordial
Primary
Preantral
Antral
Corpora Lutea
Follicle Stages
WT
mdr1a -/-

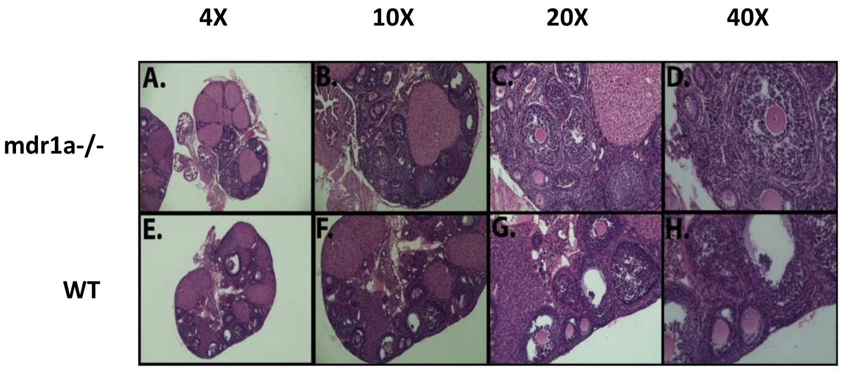
4X
10X
20X
40X
mdr1a-/-
WT
A.
B.
C.
D.
E.
F.
G.
H.

Figure S4

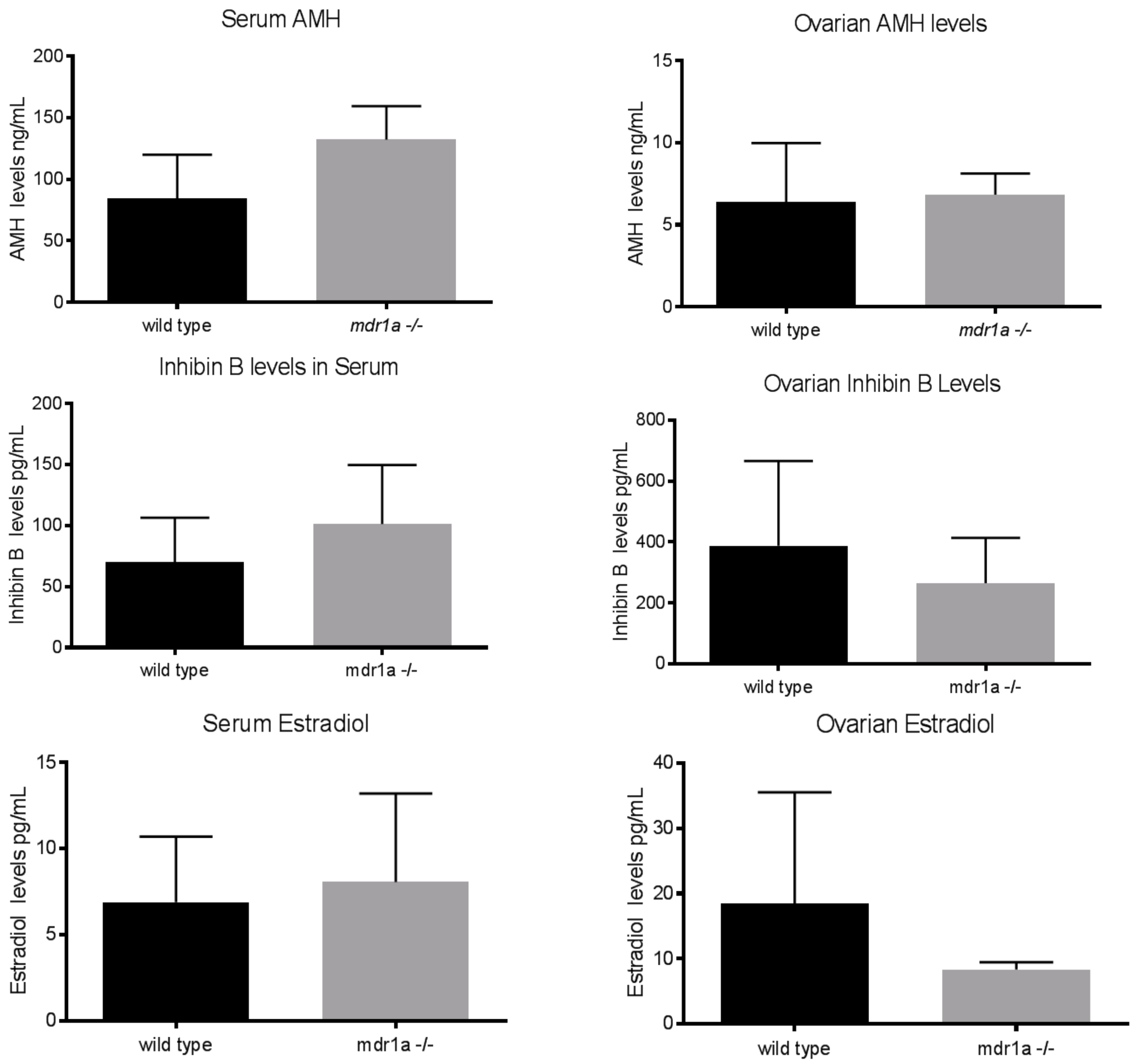

Serum AMH
AMH levels ng/mL
200
150
100
50
0
wild type
mdr1a -/-
Ovarian AMH levels
AMH levels ng/mL
15
10
5
0
wild type
mdr1a -/-
Inhibin B levels in Serum
Inhibin B levels pg/mL
200
150
100
50
0
wild type
mdr1a -/-
Ovarian Inhibin B Levels
Inhibin B levels pg/mL
800
600
400
200
0
wild type
mdr1a -/-
Serum Estradiol
Estradiol levels pg/mL
15
10
5
0
wild type
mdr1a -/-
Ovarian Estradiol
Estradiol levels pg/mL
40
30
20
10
0
wild type
mdr1a -/-

Figure S5

Genotype	Sex	Number of Mice	Instances of Tumor Occurrence	Rate of Tumor Occurrence
wild type	Female	80	0	0%
	Male	78	0	0%
	Total	158	0	0%
mdr1a-/-	Female	110	4	3.64%
	Male	79	1	1.27%
	Total	189	5	2.65%

Figure S6

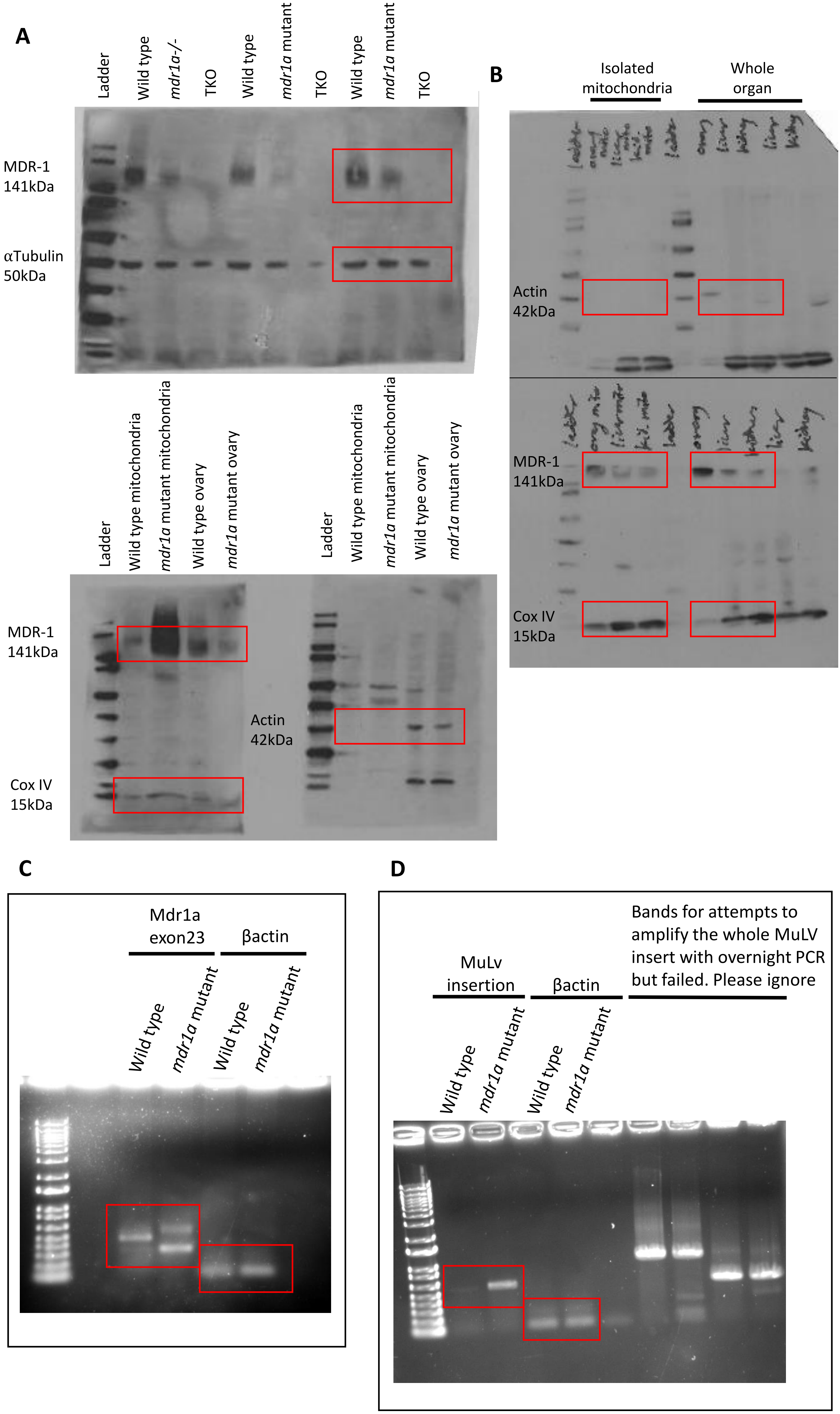

A
Ladder
Wild type
mdr1a-/-
TKO
Wild type
mdr1a mutant
TKO
Wild type
mdr1a mutant
TKO
MDR-1
141kDa
αTubulin
50kDa
Ladder
Wild type mitochondria
mdr1a mutant mitochondria
Wild type ovary
mdr1a mutant ovary
Ladder
Wild type mitochondria
mdr1a mutant mitochondria
Wild type ovary
mdr1a mutant ovary
MDR-1
141kDa
Cox IV
15kDa
Actin
42kDa
B
Isolated mitochondria
Whole organ
Actin
42kDa
MDR-1
141kDa
Cox IV
15kDa
C
Mdr1a exon23
βactin
Wild type
mdr1a mutant
Wild type
mdr1a mutant
D
MuLv insertion
βactin
Bands for attempts to amplify the whole MuLV insert with overnight PCR but failed. Please ignore
Wild type
mdr1a mutant
Wild type
mdr1a mutant

Table S1

Table S1. List of primers	
**Primer name**	**Primer sequence**
F1	ATCATTGCGATAGCAGGAGTGG
R1	CGTGCTGTAGCTGTCAATCT
F2	ATCAGCGAGACCACGATTC
R2	TGAGTTGTTGTGTCACCAAGTA
acsf2 F	TGAGCTGACCAACCTGAAC
acsf2 R	AGAAGCCCTGTTCATCCATC
c1rb F	AAATCAGGCGCTACTGTCC
c1rb R	CTGGATACTGGCTTCATAGGTG
mid1 F	CAACATCAAGCAGAACCACTAC
mid1 R	CTTGCGATGAGCCGATTTG
zbtb8b F	TCAGAACGACTGGTATAGAGAGG
zbtb8b R	GGATGTGCCGTTTGAGGAT
gulo F	CAGCCACAAGATCTTCTCCTAC
gulo R	CTCAGCAGGATGTCATCACC
tspan11 F	TTCAAATGCTGTGGCAGTAAC
tspan11 R	CCCTCTACCTTGTAGATGTTGG
mt16S F	CCGCAAGGGAAAGATGAAAGAC
mt16S R	TCGTTTGGTTTCGGGGTTTC
mtND1 F	CTAGCAGAAACAAACCGGGC
mtND1 R	CCGGCTGCGTATTCTACGTT
b-actin F	TCTTGGGTATGGAATCCTGTGG
b-actin R	CAGCACTGTGTTGGCATAGAGG
